# Injection Molding of Highly Filled Polypropylene-based Biocomposites. Buckwheat Husk and Wood Flour Filler: A Comparison of Agricultural and Wood Industry Waste Utilization

**DOI:** 10.3390/polym11111881

**Published:** 2019-11-14

**Authors:** Jacek Andrzejewski, Mateusz Barczewski, Marek Szostak

**Affiliations:** Polymer Processing Division, Institute of Materials Technology, Poznan University of Technology, Piotrowo 3 Street, 61-138 Poznan, Poland; mateusz.barczewsi@put.poznan.pl

**Keywords:** biocomposite, polypropylene, particle reinforcement, injection molding

## Abstract

The study presents a comparative analysis for two types of polymer fillers used during the processing of polypropylene by the injection molding technique. The aim of the study was to assess the usefulness of buckwheat husk waste as an alternative to the widely used wood fiber fillers. For this purpose, we prepared composite samples containing 10, 30 and 50 wt % of the filler, which were subjected to mechanical tests, thermal analysis, and structural observations in order to evaluate and compare their properties. Additionally, we evaluated the effectiveness of the composite system’s compatibility by using maleic anhydride grafted polypropylene (PP-g-MA). The results of mechanical tests confirmed a more effective reinforcement mechanism for wood fibers; however, with the addition of PP-g-MA compatibilizer, these differences were significantly reduced: we observed a 14% drop for tensile modulus and 5% for strength. This suggests high susceptibility to this type of adhesion promoter, also confirmed by SEM observations. The paper also discusses rheological measurements conducted on a rotational rheometer, which allowed to confirm more favorable flow characteristics for composites based on buckwheat husks.

## 1. Introduction

In the existing industrial practice, fibrous forms of natural fillers, which are an ecological alternative to synthetic fibers (glass, basalt, carbon, etc.) have many practical applications. In the case of cured resin processing technology, such as the RTM technique (resin transfer molding), resin infusion or compression molding [1,2,3], the fibrous form of natural fillers facilitates their use as a composite reinforcement. However, due to the dynamic development of extrusion and injection techniques, currently the greatest implementation potential is observed for composite systems based on thermoplastics matrices reinforced with short fibers [4,5]. This trend also applies to composites based on natural fillers [6,7,8], where currently conducted development works cover a wide field of research directions, aimed at improving thermo-mechanical properties, or optimizing the economic and ecological balance for prepared materials [9].The widespread use of wood industry waste and the growing availability of WPC (wood polymer composite) products is an excellent example of post-industrial waste management, leading to their quantitative reduction and the creation of added value through their use as an alternative to synthetic polymer fillers.

The need to look for a cheap and easily available source of lignocellulosic waste is the natural consequence of the increased interest in composite products of this type. From this point of view, crop plants waste, whose previous utilization consisted mainly in using it as an alternative source of energy in combustion processes, is an ideal source of raw material. Current research covering the possibility of using crop waste as composite fillers covers a very wide range of products such as straws [10,11], leaves [12,13], shells [14,15,16,17,18,19], hulls and husks [3,20,21,22]. The use of buckwheat husk discussed in this paper is one of many examples of research into utilization of waste generated during the processing of cereal grains. The selection of buckwheat (*Fagopyrum esculentum*) grain waste as the main subject of research is closely related to the great popularity of this crop in Europe. While the main research trend in this area is devoted to the use of rice and wheat husk, which is related to the dominance of these crops in the world, numerous examples of research also include the potential use for corn and soy husk [20,23,24]. It should be added that material research including the effective use of grain waste is also conducted in the direction of adopting these materials as a source of silica [25,26], nano-cellulose [27,28,29] and biochar [30,31], which can also be used as fillers in selected matrices.

The presented research offers a comparative analysis of two types of natural fillers. The reference material is a fibrous filler in the form of wood particles ground to a fine dispersive flour, a by-product of the wood industry, which for many years, has been used in the production of extruded wood-like profiles. WPC profile extrusion technology allows the use of up to 80% filler [32]. However, optimal properties are most often obtained at contents of 50% to 60% [33,34,35,36]. The polymer matrix for most of such materials is polyethylene [37,38], polyvinyl chloride [39,40] or polypropylene [41,42], the latter being the most commonly used matrix for injection molded WPC composites; therefore, it has been used in the presented studies.

The main subject of the present study is the use of grounded buckwheat husk, which is a waste product of the agri-food industry. In the current practice, the management of this type of materials includes, among others, using it as a filler for pillows and mattresses, which is an alternative for people allergic to bird feathers. In the current literature, there are no references to the use of buckwheat husk waste as fillers of polymer composites; however, the application of other types of grain husksis already a widely known issue and is currently the subject of intensive application research [37,43].

In the case of the injection technique, the use of highly filled composites encounters serious limitations connected to the necessity to ensure a sufficiently low viscosity. As a consequence, the maximum filler content used in polymer compounds dedicated to the injection molding industry rarely exceeds 40% [10,44]; however, in some studies, the filler content reaches even 60%–70% [45]. Another reason for using a reduced amount of the filler is the need to optimize the mechanical properties of products, because due to operating conditions, injection molded parts are most often subjected to dynamic loads, as opposed to extruded profiles, where static loads dominate.

The present work aims at assessing the possibility of using waste natural fillers based on buckwheat husk, as compared to commonly used wood fibers. Comparative studies of a similar type have been carried out repeatedly [46,47], but they have never included the use of buckwheat husk particles. The research also covers an evaluation of the effectiveness of interface compatibilization methods, by using the maleic anhydride grafted polypropylene PP-g-MA (commonly used additive in WPC industry). Material tests included static tensile measurements and DMTA tests. The analysis is supplemented by DSC and rheological measurements, structural changes are depicted using scanning electron microscopy (SEM) and polarized optical microscopy (POM) techniques.

## 2. Materials and Methods

### 2.1. Materials

Isotactic polypropylene HP456J from Basell Orlen Petrochemicals (Plock, Poland) (MFI= 3.4 g/10 min; 230 °C/2.16 kg) was used as the matrix material for the purpose of the study. In order to increase the adhesion coefficient samples were modified with PP-g-MA. We used PP-g-MA resin Orevac CA100 (Arkema, Colombes, France), MFI=10 g/10 min; 230 °C/0.325 kg, grafting level 1.0% MA. To evaluate the efficiency of the compatibilization procedure reference, samples were prepared without the addition of PP-g-MA compound, for the modified samples the PP-g-MA content was 5 wt %. 

Two types of natural fillers were used in the study. Buckwheat husk (BH) was the main investigated material; the purchased husk material was milled on laboratory grinder with the mesh diameter of 1 mm, material was supplied by the company First Polska (Wroclaw, Poland). The second type of filler was soft wood in the form of wood flour (WF). The used type of particles was Lignocel C120 (Rettenmaier and SohneGmbh, Rosenberg, Germany) obtained from conifer trees, the particle size ranged from 70 to 150 μm. This kind of wood flour has been used several times in combination with thermoplastic polymers [48,49,50]. More detailed description of the fillers is presented in the Supporting Information section, where the general appearance of the fillers and their microscopic images are presented (Figures S1 and S2). The particle size distribution for the buckwheat husk particles is shown in Figure S3, while the thermal stability of both fillers in the form of thermogravimetric measurements (TGA 209 Libra, Netzsch, Selb, Germany) is compared in Figure S4. The general view of the samples is shown in Figure S5. The density of the fillers was 1.471 and 1.422 g/cm^3^, for WF and BH, respectively. The density measurements were performed with the use of helium pycnometer.

### 2.2. Sample Preparation

Four types of samples were prepared for the study. The first group was filled with buckwheat husk, the second one was filled with wood flour, and the corresponding samples—with the addition of PP-g-MA. To prepare the necessary samples, all four types of composite mixtures were extruded and injection molded.

The first step of preparation consisted of material milling, which was performed using a high-speed mill (Retsch ZM200, Haan, Germany). Next, the matrix compound and the appropriate filler were mixed together using the rotational mixer (Retsch GM200). The concentration of natural filler was 10, 30 and 50 wt % by weight. The received mixture was dried before the next processing step using a cabinet dryer (24 h at 80 °C). In order to investigate the effect of the compatibilizer, the polypropylene matrix was blended with a PP-g-MA modifier. The extrusion process was performed using a twin-screw extruder (ZAMAK EH16D, Zamak Mercator, Skawina, Poland), the extruded strand was cooled in a water bath and granulated. The prepared pellets were dried before injection molding for 24 h in 80 °C. The final sample preparation was performed on the injection molding machine (ENGEL ES 80/20 HLS, Engel Austria GmbH, Schwertberg, Austria). The full list of prepared composite formulations is presented in Table 1, while detailed parameters of the extrusion and injection molding process are listed in the Supporting Information section in Table S1.

### 2.3. Material Characterization

The static tension test was conducted using the universal testing machine INSTRON 4481 (Instron, Norwood, MA, USA). The measurements were performed according to ISO 527-2 standard. The used sample type was 1A, crosshead speed was 10 mm/min, while the modulus test speed was 1 mm/min.

Izod impact tests were performed using a pendulum testing hammer Ceast 9050 (Instron, Norwood, MA, USA) in accordance with ISO 180 standard, the machine was equipped with 5 J hammer. The samples were cut out from dumbbell specimens and notched immediately after molding. Notch depth was 2 mm, while the sample dimension 10 mm × 4 mm × 60 mm. Before testing, the samples were conditioned for 48 h at room temperature.

Dynamic thermo-mechanical analysis (DMTA) was conducted with the use of Anton Paar MCR301 rheometer (Graz, Austria), equipped with a torsion clamp system, the frequency of 1 Hz and strain amplitude of 0.01% were applied. All the measurements were performed in the range of −50 to 150 °C and the heating rate was 2 °C/min. The samples for the test were cut out from the injection molded dumbbell samples, each measuring 50 mm × 10 mm × 4 mm.

In order to investigate thermal characteristic of the prepared composites, DSC measurements were performed. Single measurement consisting of standard heating/cooling/heating procedure was performed in the temperature range from 30 to 230 °C, with the heating/cooling rate of 10 °C/min. The samples were cut out from the center section of the dumbbell specimen and placed in an aluminum crucible. The average sample size was about 5 mg. All measurements were performed under protective nitrogen atmosphere. The apparatus type used was DSC F1 Phoenix (Netzsch, Selb, Germany). The crystallinity level of the composites was calculated based on the following equation:

(1) $$\%\;Crystallinity=X_{c}=\;100\;\times \;\frac{\Delta H_{m}}{\Delta H_{\mathrm{PP}}\;\left(1-\varphi \right)}$$

where Δ*H*_m_ is the measured melting enthalpy, Δ*H*_PP_ is the theoretical melting enthalpy of 100% crystalline PP (207 J/g) [51], *φ* expressed the weight percentage of used filler.

The polarized optical microscopy imaging was performed using the Nicon Eclipse E400 microscope attached with hot-stage Linkam THMS600 (Tadworth, UK). A thin, 20 µm slice of material was cut from the injection molded specimen using a microtome blade, the sampling zone was located in the middle of the dumbbell sample. Next, the specimen was placed between two glass cover slips and placed on the surface of the heated stage. The sample was heated to 200 °C and maintained at this temperature for 5 min in order to melt the matrix polymer and erase the thermal history of the sample. The isothermal crystallization step was performed at 135 °C after cooling from the melt.

The microstructural analysis of the fracture surface of the composites was conducted using a scanning electron microscope—Carl Zeiss EVO 40 (Jena, Germany). The samples were initially freeze-fractured under liquid nitrogen and then the fractured surface was coated with a fine layer of gold before observation. For the SEM investigation, the samples with filler content 10 and 50 wt % were selected, both with buckwheat husk and wood flour, in addition to the corresponding samples with maleic anhydride grafted polypropylene compatibilizer (PP-g-MA).

Rheological measurements were performed with the use of an Anton Paar MCR301 rotational rheometer, equipped with a parallel plate system (plate diameter = 25 mm). All measurements were performed in a small amplitude oscillatory shear mode at constant temperature of 190 °C and gap distance fixed at 1 mm. Strain sweep tests were performed at constant frequency ω =1 rad/s, strain amplitude range from 0.001% to 100% of strain. Frequency sweep measurements were conducted at fixed strain γ = 5% and varying angular frequency from 500 to 0.1 rad/s. During preliminary tests, the effect of the addition of PP-g-MA on rheological properties of the matrix was analyzed (see Figure S5). As was expected, very low PP-g-MA viscosity also affected the viscosity of the modified matrix, even though its percentage content reached only 5%.

## 3. Results and Discussion

### 3.1. Mechanical Properties—Static Tests and Impact Resistance

**Figure 1:**
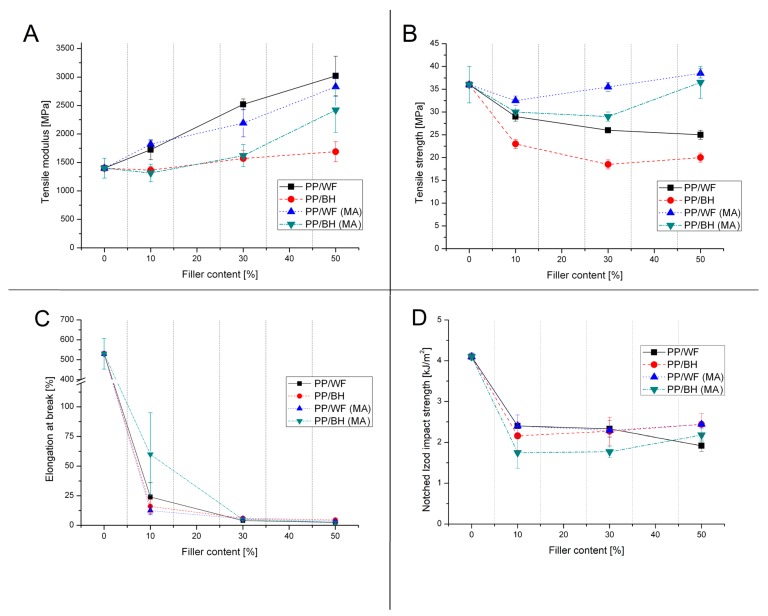
Comparison of tensile strength (**A**) and modulus (**B**), elongation at break value (**C**) and impact strength (**D**).

The mechanical properties of individual composites prepared in the course of the study are presented in Table S2 (Supplementary Information section), in order to show the changing trends for particular material groups in detail. Most results are summarized in Figure 1, in which graphs show modifications of the tensile modulus and yield strength, as well as elongation at break and impact strength.

Considering the most commonly occurring increase of the tensile modulus for the highly filled composites, the presented results do not constitute a significant argument confirming a significant level of reinforcement. It is worth adding that the relatively low reinforcement factor is mainly related to buckwheat-husk-based materials, while the stiffness of composites based on wood flour presents a relatively satisfactory upward trend. Even in the case of 10 wt % content, the module for BH-containing composites shows a slight decrease compared to reference samples made of pure PP. Interestingly, the reduction in samples stiffness also occurs for samples modified with maleic anhydride. For unmodified samples, a further increase in the content of BH causes a slight increase in the E modulus. Serious discrepancies appear for PP-g-MA modified samples, but only in the case of the content of 50 wt % BH, where the predominance of materials modified with maleic anhydride is already significant. The difference between the value of the E modulus for the material with the addition of PP-g-MA is over 700 MPa, which, nevertheless, does not change the fact that compared to composites based on wood fibers these values are lower in the entire range of contents.

The changes observed in the values of yield strength indicate a similar mechanism of deformation for the prepared types of composites. For both buckwheat filler and wood flour, an increase in the filler content is the reason for a significant reduction in the tensile strength value. Due to the fibrous nature of wood flour particles, the dominant mechanism is the fibers pull out, which consequently limits the negative effects of poor adhesion at the matrix–filler interface. For buckwheat husk, where particle morphology is more spherical, the pure debonding mechanism results in a much more pronounced decrease in strength. Modification with maleic anhydride in both cases brings a significant improvement in strength, in particular for the BH filler, which shows a significant improvement. This result confirms the high efficiency of modifications of this type of PP-g-MA compatibilizer.

### 3.2. Thermo-Mechanical Properties—DMTA Analysis

**Figure 2:**
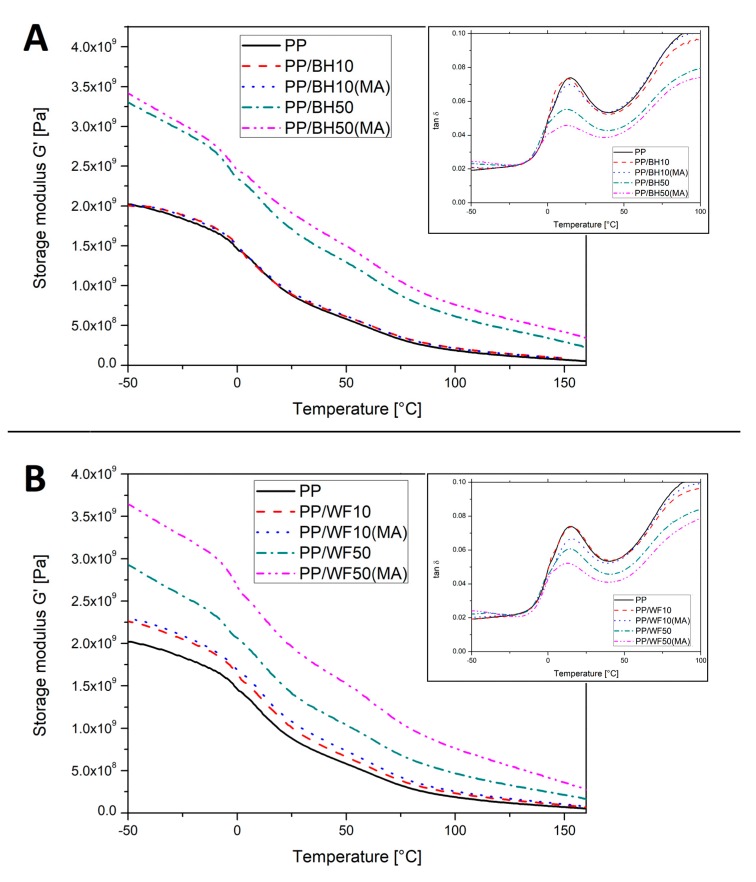
DMTA thermograms of buckwheat husk (**A**) and wood flour (**B**) based composites.

The DMTA analysis allowed for a significant extension of the temperature range of the studied visco-elastic properties changes for the obtained composites, which allowed to observe several significant differences resulting from the use of different types of reinforcing particles. The results of the measurements are presented in the form of thermograms of the storage modulus *G*′ and tan δ, separately for composites based on buckwheat husk (see Figure 2A) and wood flour (Figure 2B) are shown. The first characteristic noticeable for all the tested materials is the lack of significant change in the glass transition temperature, which can be seen on tan δ thermograms, where the main relaxation peak for polypropylene occurs for all samples in a fairly narrow temperature range of 12–15 °C. Significant differences regarding the level of material reinforcement occur on the storage modulus thermograms, the plot comparison shows that the changes in filler content from 10 to 50 wt % and the use of a PP-g-MA compatibilizer cause different behavior of composites based on buckwheat husk and wood flour. At the lowest filler level of 10 wt %, for composites based on milled husk, the course of the of *G*′ curve indicates the lack of an effective reinforcing mechanism, both for unmodified samples and those with the addition of maleic anhydride. 

Similar conclusions can be made based on the analysis of tan δ thermograms, but it should be noted that a slight reduction in the peak area for the PP/BH10(MA) sample suggests some improvement in the compatibility of the matrix–filler interactions by the appearance of chemical bonds at the interface. At the same low filler content for samples with the addition of wood flour, there is a clear reinforcement effect, which is confirmed by the increase in the value of the *G*′ modulus in the entire tested temperature range. The addition of PP-g-MA further accentuates this trend, which clearly confirms the effectiveness of this compatibilization method. The visible reduction of the area under the tan δ peak, while at the same time, the thermogram of the unmodified sample is almost identical to the reference sample serves as an additional confirmation of the chemical reaction that fasten polymer chains to the interface borders, which confirms the physical nature of interactions at the interface for the untreated composites.

The increase in the amount of filler to a maximum of 50 wt % again highlights some differences in the reinforcing mechanism for both types of the tested composites. In the case of BH-based composites, the course of the storage modulus thermograms indicates a significant increase in its values throughout the entire temperature range. The use of a PP-g-MA compatibilizer additionally improved the stiffness of the composite. This change has a relatively small absolute value; however, it confirms the effectiveness of maleic anhydride addition at the interface.

The importance of the chemical modification is more pronounced for composites based on wood flour, where significant difference in the value of storage modulus appeared for the sample with the addition of PP-g-MA and without it. Although the values of the module for the PP/WF50 sample are much higher than for the samples with the lowest 10 wt % filler content, the stiffness of the corresponding unmodified sample with the addition of 50 wt % buckwheat husk is visibly higher. This difference would indicate a more favorable reinforcing mechanism for the BH particles, which is impossible considering the much more favorable fibrous morphology and homogenous particle size distribution for wood flour. The main reason for these reduced values of *G*′ modulus is the agglomeration of wood fibers, leading to a significant reduction in the effective surface interactions at the matrix–filler interface. This conclusion is confirmed by the tan δ curve analysis, where the reduction of the relaxation peak area is relatively small considering the amount of the filler. Modification with maleic anhydride eliminates the issue of fibers agglomeration, which clearly increases the performance of the tested composites. In the case of PP/WF50(MA) samples, the value of the storage module achieves its maximum in the entire tested temperature range.

### 3.3. Nucleation of the Crystalline Phase and Analysis of Spherulites Growth Kinetics—DSC Analysis, POM Observations

**Figure 3:**
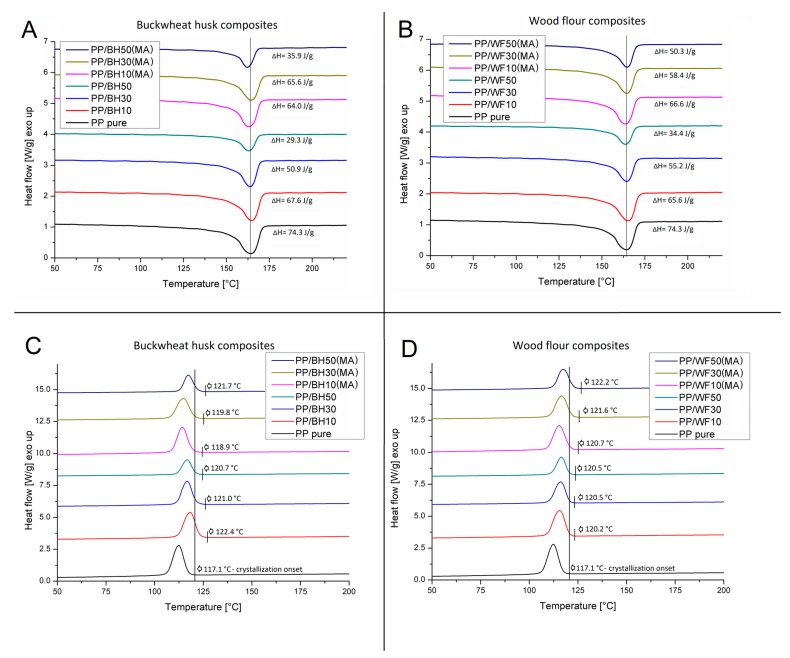
Comparison of the heating thermograms for buckwheat husk (**A**) and wood flour (**B**) composites, and cooling thermograms of the buckwheat husk (**C**) and wood flour (**D**) composites.

**Figure 4:**
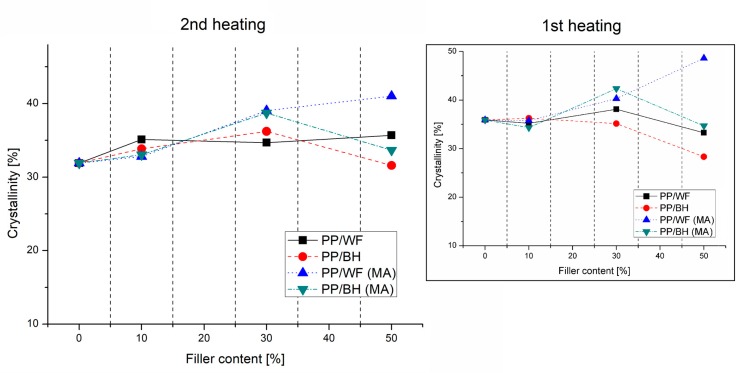
Crystallinity level calculated from the 1st and 2nd heating cycle of the DSC measurements.

Thermal properties of the prepared materials were analyzed on the basis of DSC measurements conducted in the course of the standard heating/cooling/heating procedure. For the purpose of comparison, thermograms of the second heating are shown in Figure 3, while the graphs presented in Figure 4 present a summary of the results of crystallinity measurements conducted during both heating cycles.

The comparison of the heating thermograms does not reveal any significant differences between the prepared composites. The position of the melting peak for all the tested samples is close to 165 °C, which corresponds to the reference PP sample. Changes in the surface area of the melting enthalpy also do not indicate any significant changes in the thermal properties of the tested samples, while the change in their values, marked on the graph, are mainly related to the variation of the matrix/polymer ratio content for individual samples. The crystallinity level of the PP matrix was calculated from both heating cycles. For the samples with the lowest filler content, the amount of the PP crystalline phase was close to the results obtained for the reference sample; however, an increase in the amount of reinforcement to 30 wt % revealed visible discrepancies compared to pure polypropylene. In the case of both types of unmodified samples, the content of the crystalline phase remained at the reference level, while the addition of PP-g-MA indicated a certain upward trend for both WF and BH filler, which can be recorded for both heating cycles.

For samples with the highest content of the fillers in their structure, the observed discrepancy in the results was high, which probably reflects significant differences in the morphology of wood and buckwheat husk particles. Wood fibers have a more favorable morphology due to the much larger area of surface interaction. This leads to an increase in efficiency of the nucleation phenomena, in particular, the most evident case was for the samples modified with maleic anhydride, the presence of which leads to an additional improvement in the dispersion of wood fibers. Meanwhile, for the PP/WF50 sample the matrix crystallinity remained similar to 30% WF composite and for both types of composites based on buckwheat husk, the crystalline phase content returned to the reference value of pure PP, which indirectly confirms the dominant physical nature of interactions at the matrix–filler interface. The results for the measurements conducted in the first heating cycle are characterized by a greater dispersion of the crystallinity values, which results from unavoidable differences in the sample preparation; however, for most of the investigated composite types, the observed trends are repeatable in both heating cycles and the crystallinity of PP/WF50(MA) significantly exceeds the results obtained for other materials.

The nucleation effect can also be observed on the cooling thermograms (Figure 3C,D), in which the onset temperatures of the PP crystallization process are highlighted for all the composite samples presented on the graph. For both types of composites (filled with buckwheat husk and wood-based materials), the beginning of the exothermic peak is slightly shifted to higher temperatures in relation to the reference value of 117.1 °C obtained for pure PP. The highest temperature of the beginning of the crystallization process was recorded at 122.4 °C, for the PP/BH10 sample. However, as can be seen in the graph, there is no visible trend which would connect this phenomenon with the filler type/content or PP-g-MA modification. Considering the results obtained during DSC measurements, it can be concluded that the crystallinity of the composite matrix is only slightly dependent on the used reinforcing system, which is primarily related to the effective crystal growth mechanism of polypropylene matrix.

**Figure 5:**
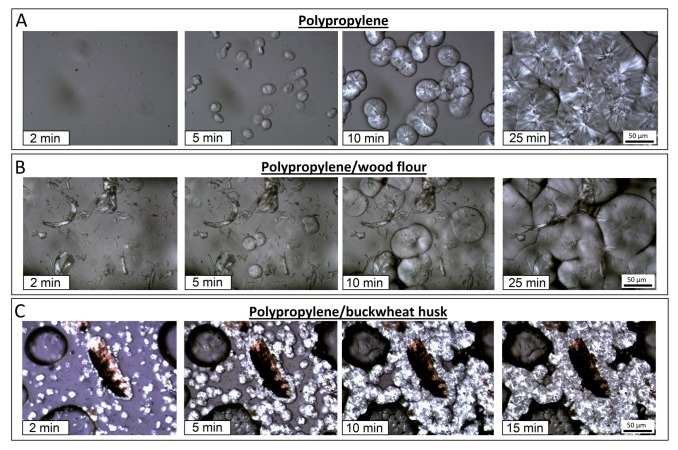
PP crystalline phase growth observed for pure PP resin (**A**), PP/wood flour composites (**B**), and buckwheat husk composites (**C**).

In order to illustrate possible differences during the growth of the PP crystalline phase, DSC studies were supplemented with optical microscope measurements. The comparison shown in Figure 5 illustrates the spherulites growth in the course of isothermal measurements performed at 135 °C. Individual series of pictures present this process for pure polypropylene (PP), sample with the addition of wood flour (PP/WF10) and buckwheat husks composite (PP/BH10). The growth of the crystalline phase both for the pure PP sample and the wood-flour-based composite is fairly slow under isothermal conditions, and an almost similar size of spherulites suggests that the surface of wood fibers does not reveal high nucleation ability of PP crystalline phase, as it was reported in the literature [52]. A different behavior was observed in the sample with the addition of buckwheat husk, where the growth of the crystalline phase spherulites was much faster and it was observed both on the surface of larger particles of the husk filler and around the smallest particles, measuring a few micrometers.

The observed spherulite growth process indicates an effective nucleation mechanism occurring on the surface of the husk particles. This process can be associated with the presence of silica in the husk cell wall structure. As indicated by most studies, in the case of grain hulls, silica concentrates mainly on the surface of the epidermis, which, in turn, increases the thermal resistance of grains preventing from excessive exposure to the sunlight [53,54]. The accumulation of inorganic constituents on the outer cell wall of the husk compensates for the relatively low percentage of silica in the hull structure (about 1.7%), and also visibly modifies the kinetics of the growth of the PP crystalline phase. A similar behavior is observed when using silica in its pure form obtained by treating the rice husk [25], where the crystals growth kinetic is also accelerated, but again, this does not significantly increase the amount of crystalline phase.

### 3.4. Morphological Analysis—BH and WF Composites Structure Comparison

**Figure 6:**
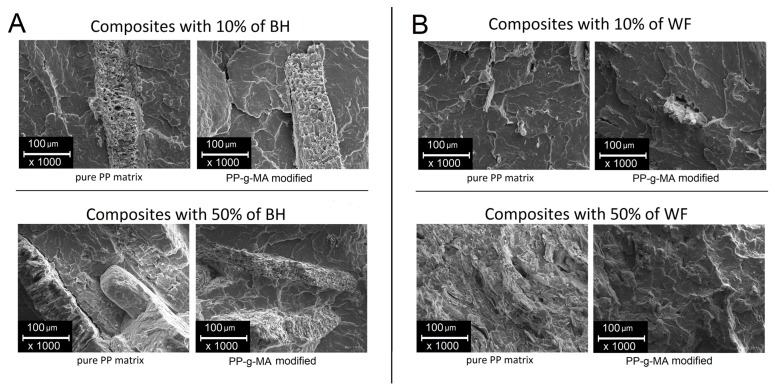
Buckwheat husk-based composites, comparison of untreated and PP-g-MA modified samples (**A**), and analogous composites with an addition wood flour (**B**).

Structural analysis was carried out on samples subjected to cryo-fracturing. The subjected surface presents the structure of composites based on buckwheat husks (Figure 6A) and wood flour (Figure 6B). The first visible difference relates to the size of the individual particles, where the grounded buckwheat husk particles often cover the entire imaging surface, while the main fraction includes particles with measuring 600–1000 μm, which makes it difficult to analyze the degree of dispersion. The size of wood fibers usually does not exceed 100 μm, which facilitates observation. The appearance of unmodified composite structures for both types of fillers suggest the lack of strong interactions at the matrix–filler interface, which is particularly visible for the samples with the highest content of buckwheat husk, where the PP matrix clearly separates from the husk particles, forming a visible interface gap. The lack of compatibility is also visible for wood fibers, where the lack of chemical bonds of both phases results in the agglomeration of fibers in the PP/WF50 sample, as well as the lack of wettability of fibers through the PP matrix, which is a typical behavior for natural fillers [55]. Significant structural changes in both presented cases demonstrates the composite system compatibility with PP-g-MA. In the case of buckwheat husk particles, there is a clear increase in wettability of the filler surface, the sharp gap at the interface disappears, which, in turn, increases the effectiveness of reinforcement by limiting the phenomenon of debonding, which has the greatest impact on limiting strength for the fillers of this type. For wood fibers, an additional visible aspect of the modification with PP-g-MA is an improved dispersion of the reinforcement phase, which consequently leads to the highest stiffness for the PP/WF50(MA) samples.

### 3.5. Rheological Behavior—Influence of Filler Concentration and Compatibilization Method

**Figure 7:**
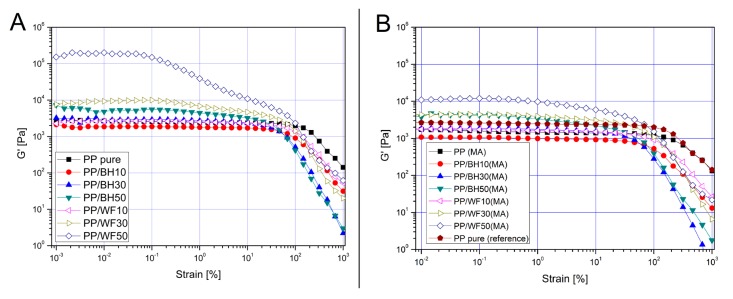
Strain sweep measurements for unmodified samples (**A**) and after addition of P-g-MA compatibilizer (**B**), ω = 1 rad/s, T = 190 °C.

**Figure 8:**
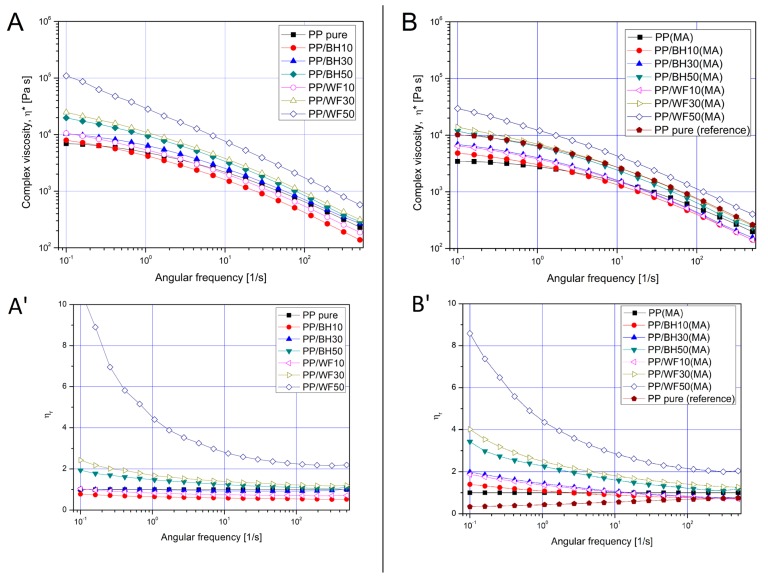
Frequency sweep measurements for unmodified samples (**A**,**A′**) and after addition of PP-g-MA compatibilizer (**B**,**B′**), γ = 5%, T=190 °C. Bottom graphs (**A′**,**B′**) presents the relative viscosity plots, which are calculated from the complex viscosity measurements presented at the top (**A**, **B**).

Rheological measurements were conducted in the dynamic mode (small amplitude oscillatory shear) on a rotational rheometer, both strain sweep and frequency sweep measurements were performed at the constant temperature of 190 °C. The graphs shown in Figure 7 present a comparison of *G*′ curves for all of composite materials, separately for the unmodified samples and for the materials compatible with PP-g-MA. The frequency sweep analysis is shown in Figure 8, where complex viscosity plots are supplemented with the relative viscosity values, calculated according to following equation:

(2) $$\eta_{r}=\eta^{*}/\eta_{PP}^{*}$$

where absolute value of complex viscosity at given frequency η* is normalized by the viscosity of pure matrix $\eta_{PP}^{*}$.

The first visible difference relates to the unusual behavior of the PP/WF50 sample, for which the *G*′ values increase significantly in almost the entire range of linear viscoelastic domain of the other samples. The reason for this behavior is the agglomeration of wood fibers observed in the previously presented results, which, in turn, leads to the appearance of mechanical interactions between the fibers of the reinforcement. The rheological behavior of molten polymer composites is strongly influenced by filler volume fraction, and similarly to other particle filler suspensions, can be classified into dilute, semidilute, concentrated isotropic and nematic regime, depending on the type of internal interactions. In the case of the discussed materials, the dilute regime refers to the BH composites with a filler content of 10 and 30 wt % and WF 10 wt % samples, both uncompatibilized and PP-g-MA modified. For these materials, there are no mechanical interactions between the individual filler particles, and moreover, hydrodynamic interactions are very limited, which can be observed both by the negligible differences in the *G*′ value on the amplitude sweep graph, and very similar values of complex viscosity in frequency sweep measurements Figure 8.

The first significant difference in rheological characteristics for BH and WF samples occurs at the filler content of 30 wt %. Despite the same filler content, the hydrodynamic interactions for wood-fiber-based composites are clearly increasing, which means that this type of material can be characterized as semidilute regime. In the discussed case, where rheological properties still do not indicate strong hydrodynamic interactions for the buckwheat husk, the main reason for the different behavior of the PP/WF30 sample is the higher aspect ratio (L/d) for WF, which according to the literature is around 10 [50,51] for this type of commercially available fibers, whereas for grounded husk fillers this coefficient ranges from 2 to 3 [56,57,58].

The analysis of rheological characteristics for the materials with the highest degree of filler loading clearly highlights the effectiveness of using PP-g-MA modifications for wood fibers. The large increase in storage modulus *G*′ values at low strains, high degree of strain softening, and no occurrence of distinct viscoelastic region clearly indicate the appearance of a strong mechanical interaction between filler particles. This means that for the PP/WF50 sample the formation of a fiber network causes the transition from semidilute to concentrated isotopic regime; some similar behavior was already reported for short glass fiber composites [4,59]. The significant increase in the complex viscosity η* (Figure 8A,B), as well as the relative viscosity *η*_r_ (Figure 8A’,B’), also indicate the appearance of additional filler–filler interactions. In the case of the samples based on buckwheat husk with the same filler content of 50 wt %, the appearance of *G*′ curve indicates that for the husk particles interactions are mainly hydrodynamic, which would indicate semidilute regime. Both plots of *G*′ and complex viscosity η* have values similar to those presented by the sample with the addition of 30 wt % WF. 

The curve analysis for PP-g-MA-modified materials indicates some significant change for the materials with the highest degree of loading. Although the PP/WF50(MA) sample is still characterized by the highest viscosity, there is a clear change in the *G*′ curve, where the value of the module decreases considerably at low strain values, and the appearance of the curve is very similar to those observed for other materials, which indicates that this material is in a semidilute regime. Such a change in rheological characteristics indicates a better dispersion of wood fibers, which results from stronger interfacial interactions and prevents the formation of agglomerates, whose presence is also the main cause of the decrease in the effectiveness of interactions in the solid state. It is worth mentioning that the frequency sweep analysis indicates that none of the tested composites shows a transition to the nematic regime (see Figures S6 and S7), the confirmation of this is the constant presence of *G*′ and *G*′′ curve intersection point, while for solid-like systems, after crossing the critical concentration level, these curves run in parallel. This fact indicates the possibility of introducing a larger amount of the filler while maintaining its concentrated isotropic character.

Summing up the conducted rheological tests, it can be concluded that from a practical point of view, the use of buckwheat hull particles has a much smaller impact on the rheological properties of the obtained composites, which consequently greatly facilitates the effective conduct of the injection molding process, especially considering that in the case of potential applications, it would be possible to obtain thin-walled products with less power consumption of injection units [60].

### 3.6. Chemical Structure Evaluation—FTIR Analysis

**Figure 9:**
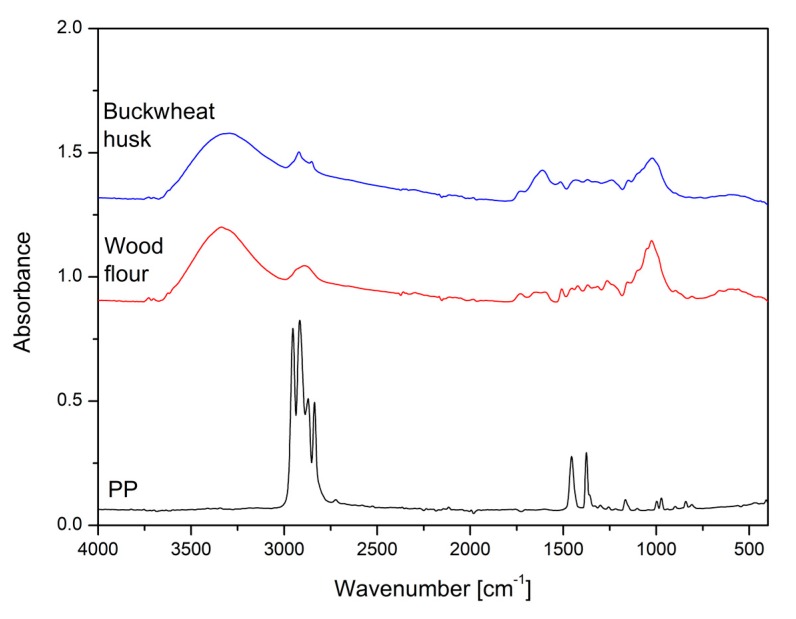
FTIR spectra of pure PP resin, wood flour (WF) and buckwheat husk (BH) filler.

IR spectroscopy was used to detect any differences in the chemical structure of the used filler components and verify the possibility of permanent chemical bonds between the filler and the matrix modified with PP-g-MA. The spectra of pure PP, BH and WF filler are presented in the Figure 9. Due to the low concentration of grafted PP (5%) the compatibilized PP/PP-g-MA blend is not presented. Taking into account the FTIR spectra of natural fillers, there are two main characteristic regions. The first one in the range between 3800 and 2700 cm^−1^ corresponds with the –OH and –CH stretching vibrations and the second one, between 1800 and 800 cm^−1^, was attributed to the different compounds of lignocellulosic fillers. FTIR spectrum analysis indicates almost identical chemical composition of both types of natural fillers. The only noticeable difference relates to the area of methyl and methylene stretching bands occurrence at 2920 and 2850 cm^−1^. For BH filler, these two bands are more pronounced. According to the literature, the difference can be attributed to the presence of organic extractives like fatty acid methyl esters and phenolic acid methyl esters [61]. Both compounds contain methyl and methylene groups; therefore, their higher content may lead to the observed spectral changes.

**Figure 10:**
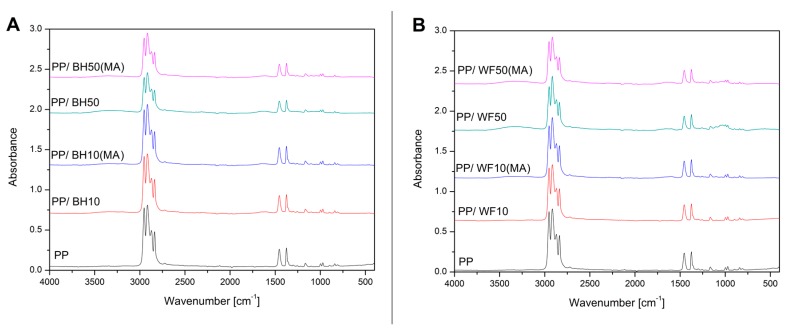
The comparison of FTIR spectra for BH- (**A**) and WF-based (**B**) composites. The plots are presenting the composite samples containing 10% and 50% of the fillers, before and after addition of PP-g-MA modifier. The reference PP sample is also presented.

The FTIR spectra made for selected polymer composites are presented in the Figure 10. Comparing to pure PP, all of the composite samples are characterized by the presence of broad peak at 3400 cm^−1^. This band can be attributed to the–OH hydroxyl group stretching, while it is more pronounced for samples containing 50% of the filler. The second of the visible differences concerns the broad peak detected at 1650–1500 cm^−1^. This band might correspond with the vinyl C=C groups at 1650 cm^−1^, or the lignin aromatic ring. The presence of these bands is caused by the increasing content of the main components of both natural fillers, including cellulose, lignin and hemicellulose. It is worth noticing that there is the lack of a clear band of C=O carbonyl groups, which could be evidence of an intensive PP degradation process.

## 4. Conclusions

Considering the potential use of natural fillers in the processing of polymer composites by the injection molding method, the use of agricultural industry waste may be an interesting alternative to wood fibers. The presented research on polypropylene-based buckwheat husk composites shows that mechanical properties do not differ significantly from those obtained with the use of wood flour, especially for high-filled composites. This is evidenced by a small drop in tensile modulus and strength values, 14% and 5%, respectively, for the highest content of the filler. As indicated by our research and the literature review, some advantage in the case of injection molding technology is manifested by much more favorable rheological characteristics of husk-based composites. The main factor that distinguishes the buckwheat husk from the used wood fibers is the smaller aspect ratio, which makes the model of behavior of this type of non-fibrous natural fillers more similar to composites based on mineral fillers such as chalk or talc. In this case, due to the lower tendency to form the filler agglomerates, the use of compatibilization methods, such as the used maleic anhydride modification, is more effective, as confirmed by most of the presented studies. Taking into account some technological issues, the necessity of proper fragmentation of the filler fraction or possible agglomeration of its particles, processing of waste in the form of grain hulls can be considered as a less demanding and more sustainable process. In summary, the research results indicate the possibility of replacing wood fibers with agricultural industry waste. In the coming years, wood fibers will continue to be the main component of natural fillers; however, due to the increasing deforestation of many regions of the earth, the price of this raw material will systematically increase, which may result in the need to use alternative materials, such as tested grain husk waste. The next research planned by our team will include a further assessment of the performance of the presented composites and the possibility of using grain husk fillers in the processing of biodegradable polymers.

## Figures and Tables

**Table 1:** Compositions of the prepared samples, density.

Sample	Matrix [wt %]		Filler [wt %]		Density * [g/cm^3^]	Void Content ** [%]
	iPP	PP-g-MA	Buckwheat Husk (BH)	Wood Flour(WF)		
**Unmodified Samples**						
PP	100	-	-	-	0.895	-
PP/WF10	90	-	-	10	0.919	1.37
PP/WF30	70	-	-	30	0.987	2.61
PP/WF50	50	-	-	50	1.075	3.36
PP/BH10	90	-	10	-	0.918	1.36
PP/BH30	70	-	30	-	0.969	4.50
PP/BH50	50	-	50	-	1.035	6.98
**Maleic Anhydride Modified Composites (PP/PP-g-MA = 95%/5%)**						
PP/WF10(MA)	85.5	4.5	-	10	0.916	1.47
PP/WF30(MA)	66.5	3.5	-	30	0.984	2.24
PP/WF50(MA)	47.5	2.5	-	50	1.062	3.27
PP/BH10(MA)	85.5	4.5	10	-	0.921	0.88
PP/BH30(MA)	66.5	3.5	30	-	0.976	3.02
PP/BH50(MA)	47.5	2.5	50	-	1.094	0.45

* sample density was measured with the use of helium pycnometer (Thermo Scientific Pycnomatic), according to ASTM 792-66 standard, standard deviation for all measurements was below 0.01 g/cm^3^. ** the void content was calculated from the theoretical and real density of the prepared samples.

## Supplementary Materials

The following are available online at https://www.mdpi.com/2073-4360/11/11/1881/s1.
