# Supplementary material for: Injection Molding of Highly Filled Polypropylene-based Biocomposites. Buckwheat Husk and Wood Flour Filler: A Comparison of Agricultural and Wood Industry Waste Utilization
Source: Polymers (Basel). 2019 Nov 14;11(11):1881. doi: 10.3390/polym11111881 (PMC6918422; doi:10.3390/polym11111881)
Supplement: Supplementary file 1 [file polymers-11-01881-s001.pdf]

Supplementary Information

Injection molding of highly filled polypropylene-based biocomposites. Buckwheat husk and wood flour filler: a comparison of agricultural and wood industry waste utilization

Jacek Andrzejewski*, Mateusz Barczewski, Marek Szostak*

Polymer Processing Division, Institute of Materials Technology, Faculty of Mechanical Engineering and Management, Poznan University of Technology, Piotrowo 3, 61-138, Poznan, Poland

*Corresponding author: jacek.andrzejewski@put.poznan.pl, marek.szostak@put.poznan.pl

Keywords: Biocomposite, Particle reinforcement, Polypropylene, Mechanical properties, Injection molding,

Natural fillers characteristics and sample appearance

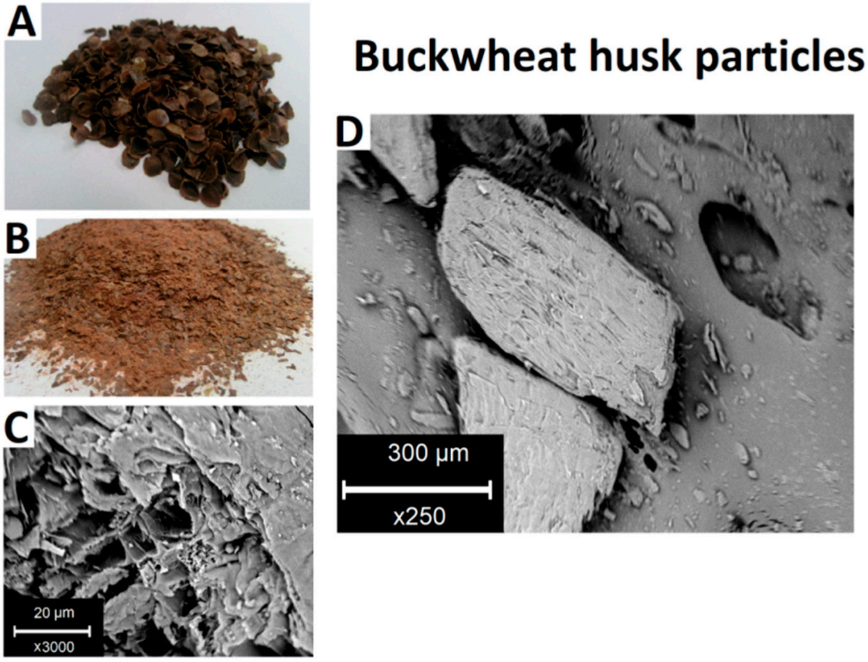

Figure S1. The appearance of buckwheat husk particles: as received (A), grounded husk (B), and SEM pictures at high and low magnification (C, D)

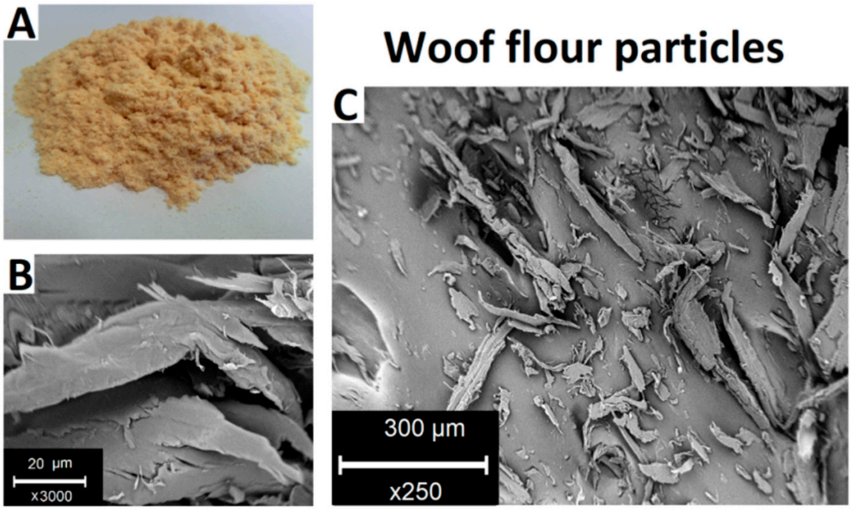

Figure S2. The appearance of wood flour particles: as received powder (A), and SEM pictures at high and low magnification (B, C)

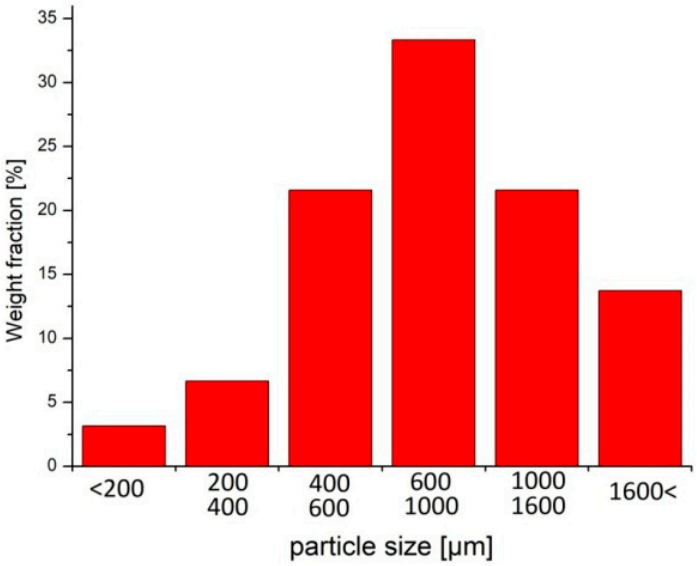

Figure S3. Particle size distribution of the grounded buckwheat husk particles. Analysis was performed using the vibratory sieve shaker Analysette 3 (Fritsch, Germany).

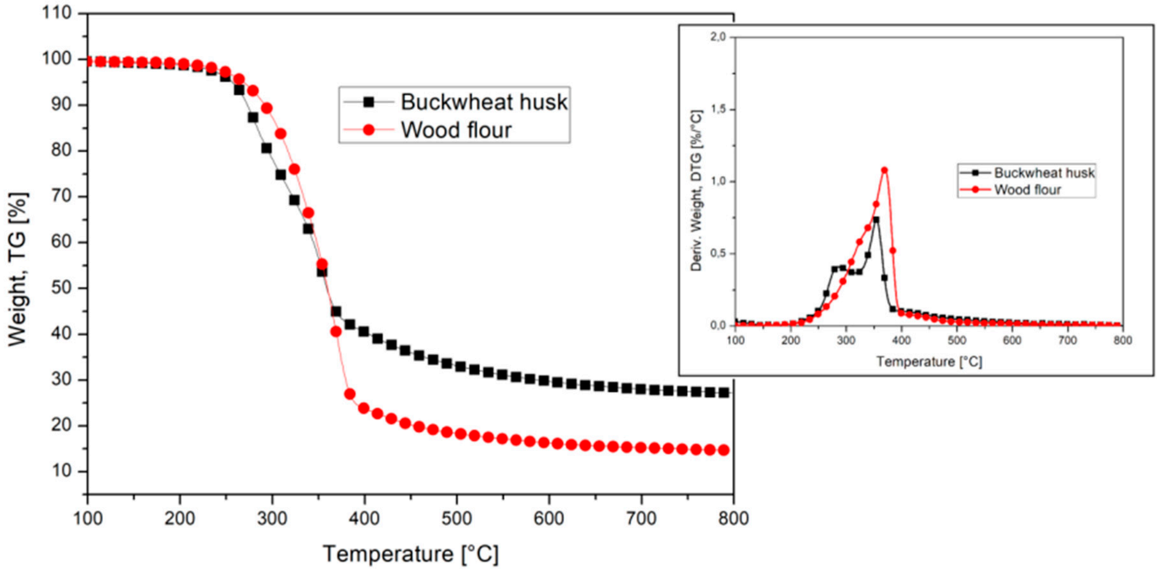

Figure S4. Thermogravimetric thermograms for BH and WF filler, the plots presents TG and corresponding DTG curves. Measurements performed under nitrogen atmosphere, heating rate 10 °C/min, temperature range 30-800°C.

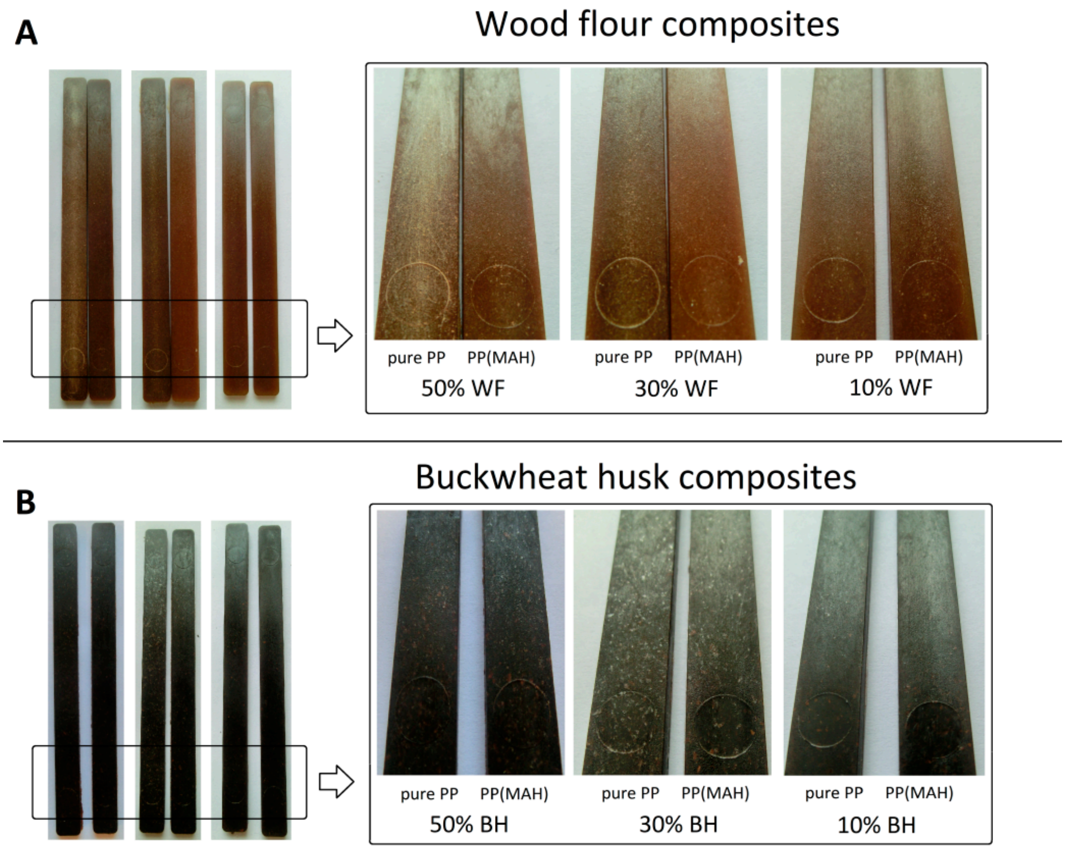

Figure S5. The appearance of the injection molded samples, rectangular bars for impact resistance tests. General view for samples with the addition of wood flour (A) and buckwheat husk (B), and magnification presenting the view of the sample surface.

Table S1. Extrusion and injection molding parameters

Parameter		
**Extrusion**		
Temperature profile	[°C]	180(die)-190-190-190-180-175-170-165-160
Screw speed	[rpm]	100
**Injection molding**		
Temperature profile	[°C]	190(nozzle)-190-190-190-180
Injection pressure	[bar]	1200
Holding pressure	[bar]	750
Holding time	[s]	5
Clamping force	[kN]	200
Cooling time	[s]	30
Screw speed	[rpm]	150
Screw back pressure	[bar]	10
Mold temperature	[°C]	40

Mechanical properties

Table S2. Mechanical test obtained from the static tensile test and Izod notched impact test

Sample	Static tensile test				Izod test
	Tensile Modulus [MPa]	Tensile Strength [MPa]	Elongation at yield [%]	Elongation at break [%]	Impact strength [kJ/m^2]
**Unmodified samples**					
PP	**1400** (±176)	**36.0** (±4.0)	**11.0** (±1.5)	**530.0** (±77)	**4.1** (±0.1)
PP/ WF10	**1725** (±175)	**29.0** (±1.0)	**5.0** (±0.5)	**24.0** (±12.0)	**2.4** (±0.1)
PP/ WF30	**2520** (±95)	**26.0** (±0.5)	**2.5** (±0.3)	**4.0** (±0.7)	**2.3** (±0.2)
PP/ WF50	**3020** (±345)	**25.0** (±1.0)	**2.0** (±0.2)	**2.5** (±0.4)	**1.9** (±0.1)
PP/ BH10	**1365** (±65)	**23.0** (±1.0)	**5.0** (±0.5)	**16.0** (±6.0)	**2.1** (±0.3)
PP/ BH30	**1570** (±145)	**18.5** (±1.0)	**3.0** (±0.5)	**6.0** (±1.5)	**2.3** (±0.3)
PP/ BH50	**1690** (±175)	**20.0** (±1.0)	**2.5** (±0.4)	**4.5** (±1.0)	**2.4** (±0,3)
**MAPP modified composites**					
PP/ WF10(MAH)	**1820**(±65)	**32.5** (±1.0)	**5.0** (±0.5)	**12.5** (±3.5)	**2.4** (±0.3)
PP/ WF30(MAH)	**2190** (±240)	**35.5** (±1.0)	**3.5** (±0.2)	**5.5** (±0.6)	**2.3** (±0.1)
PP/ WF50(MAH)	**2830** (±170)	**38.5** (±1.0)	**3.0** (±0.2)	**3.5** (±0.4)	**2.4** (±0.1)
PP/ BH10(MAH)	**1315** (±155)	**30.0** (±0.5)	**6.5** (±1.0)	**60.0** (±35.0)	**1.7** (±0.4)
PP/ BH30(MAH)	**1620** (±195)	**29.0** (±1.0)	**3.5** (±0.2)	**5.0** (±1.0)	**1.8** (±0.1)
PP/ BH50(MAH)	**2420** (±390)	**36.5** (±3.5)	**2.5** (±0.2)	**2.5** (±0.2)	**2.2** (±0.1)

Rheological analysis

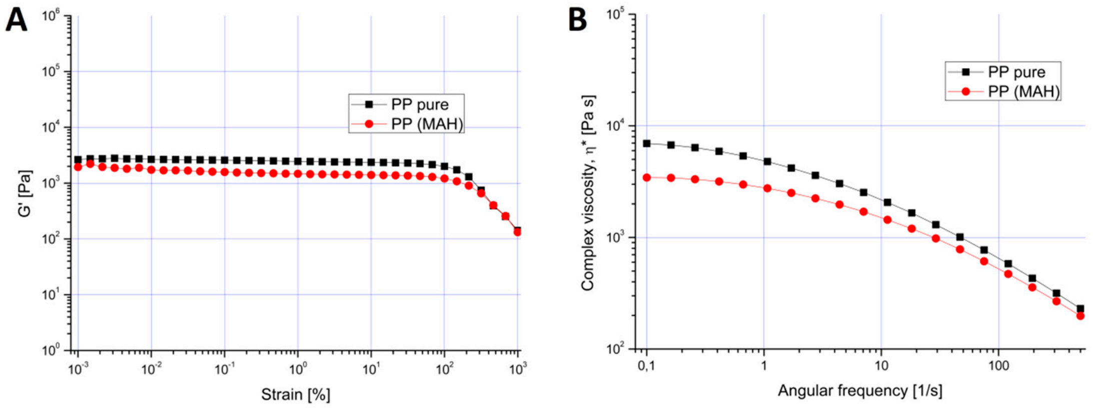

Figure S6. Comparison of storage modulus G' (A) and complex viscosity η* (B) curves for pure PP resin and MAH modified polypropylene. Strain sweep measurements were performed at constant deformation frequency ω=1 rad/s, while frequency sweep test at constant strain γ=5 %.

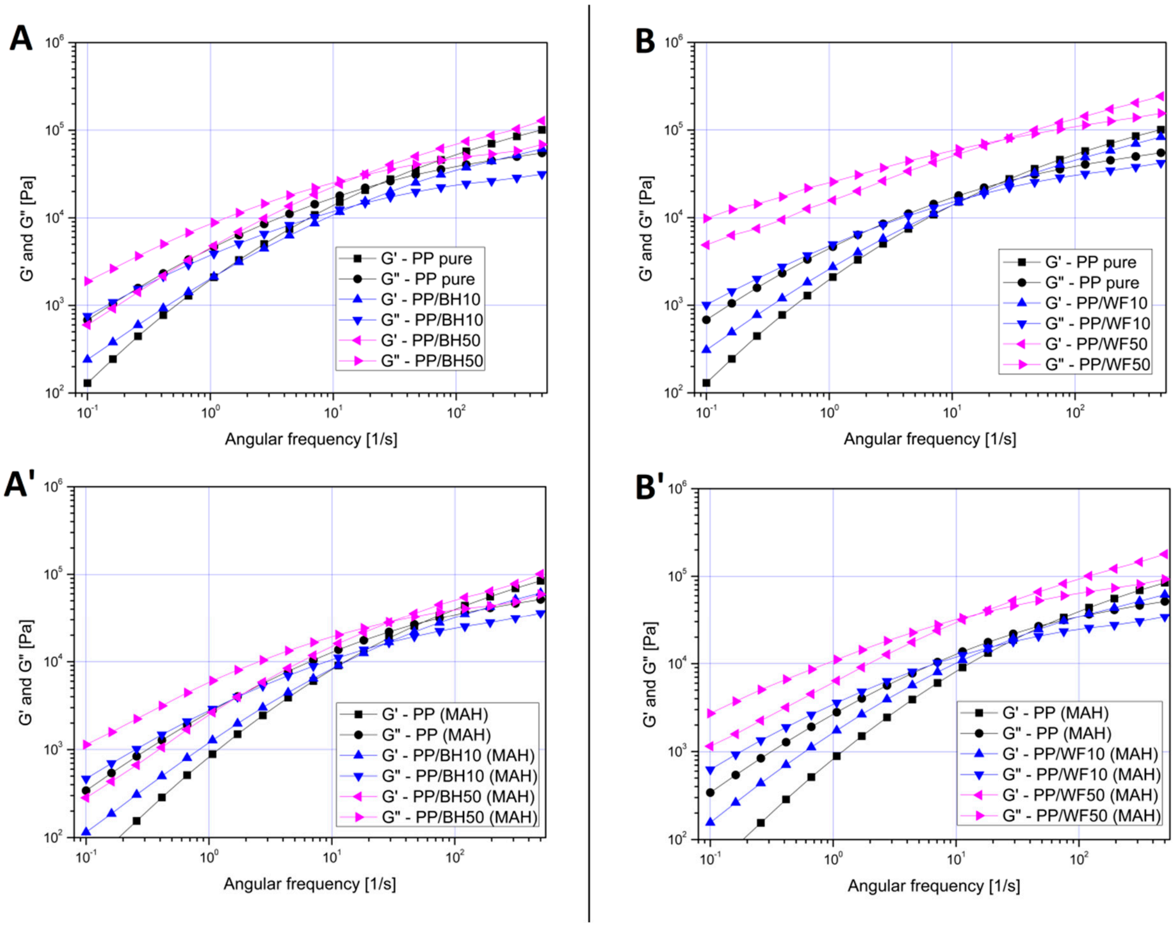

Figure S7. Frequency sweep analysis, comparison of G' and G" plots buckwheat husk and wood flour-based composites: unmodified (A, B), and after MAH addition (A', B'). For clarity, the graphs present the results for matrix resin and samples with 10 and 50 % filler content.
